# Impact of maternal immune activation and sex on placental and fetal brain cytokine and gene expression profiles in a preclinical model of neurodevelopmental disorders

**DOI:** 10.1186/s12974-024-03106-7

**Published:** 2024-05-07

**Authors:** Hadley C. Osman, Rachel Moreno, Destanie Rose, Megan E. Rowland, Annie Vogel Ciernia, Paul Ashwood

**Affiliations:** 1 Department of Medical Microbiology and Immunology, University of California, Davis, Davis, CA USA; 2 The M.I.N.D. Institute, University of California at Sacramento, Sacramento, CA USA; 3 Department of Biochemistry and Molecular Biology, University of British Columbia, Vancouver, Canada; 4 Djavad Mowafaghian Centre for Brain Health, University of British Columbia, Vancouver, Canada

**Keywords:** Maternal immune activation, MIA, Placenta, Fetal brain, Cytokines, Synapse, Development, Neurodevelopment, Autism, Autism spectrum disorder (ASD), Schizophrenia

## Abstract

Maternal inflammation during gestation is associated with a later diagnosis of neurodevelopmental disorders including autism spectrum disorder (ASD). However, the specific impact of maternal immune activation (MIA) on placental and fetal brain development remains insufficiently understood. This study aimed to investigate the effects of MIA by analyzing placental and brain tissues obtained from the offspring of pregnant C57BL/6 dams exposed to polyinosinic: polycytidylic acid (poly I: C) on embryonic day 12.5. Cytokine and mRNA content in the placenta and brain tissues were assessed using multiplex cytokine assays and bulk-RNA sequencing on embryonic day 17.5. In the placenta, male MIA offspring exhibited higher levels of GM-CSF, IL-6, TNFα, and LT-α, but there were no differences in female MIA offspring. Furthermore, differentially expressed genes (DEG) in the placental tissues of MIA offspring were found to be enriched in processes related to synaptic vesicles and neuronal development. Placental mRNA from male and female MIA offspring were both enriched in synaptic and neuronal development terms, whereas females were also enriched for terms related to excitatory and inhibitory signaling. In the fetal brain of MIA offspring, increased levels of IL-28B and IL-25 were observed with male MIA offspring and increased levels of LT-α were observed in the female offspring. Notably, we identified few stable MIA fetal brain DEG, with no male specific difference whereas females had DEG related to immune cytokine signaling. Overall, these findings support the hypothesis that MIA contributes to the sex- specific abnormalities observed in ASD, possibly through altered neuron developed from exposure to inflammatory cytokines. Future research should aim to investigate how interactions between the placenta and fetal brain contribute to altered neuronal development in the context of MIA.

**Supplementary Information:**

The online version contains supplementary material available at 10.1186/s12974-024-03106-7.

## Introduction

Autism spectrum disorder (ASD) is a neurodevelopmental condition that presents with repetitive behaviors and social impairment in diagnosed individuals [43]. As of 2020, 1 in 36 children are diagnosed with ASD by the age of 8 in the United States [46]. ASD is more common in males than females, with a ratio of 4:1, suggesting that there are sex-dependent variables at play [19, 70]. The exact etiology for many ASD cases is largely unknown, however, genetic and environmental risk factors are thought to contribute to its pathophysiology. There are hundreds of heritable and *de novo* single gene variants that have been linked with ASD [2]. However, the presence of common variant ASD risk genes *per se*, is not an absolute indicator that an individual will develop ASD [22]. More recently, the attention has shifted towards the interactions of genes and the analysis of gene pathways, as well as epigenetic and environmental factors that may influence the transcriptome [2, 61].

Evidence suggests that environmental factors likely play a major role in the development of ASD. During development, exposure to toxins or disruption of the immune system may alter brain structure and function and lead to neurodevelopmental disorders (NDD) [24, 59]. Inflammation during pregnancy, whether due to infection, exposure to allergens or pollution, or inflammatory disease, is associated with a higher risk of NDD in the offspring [31, 31, 53]. Epidemiological studies have shown that women hospitalized with an infection during pregnancy have an increased likelihood of having a child with ASD [5, 34]. Similarly, animal models of maternal infection using polyinosinic: polycytidylic acid (poly (I: C)) or lipopolysaccharide (LPS) have shown altered behavior and brain development in the offspring [15, 27, 31, 32, 58, 60].

Cytokines are immune signaling molecules that play a major role in the inflammatory response. More recently, cytokines have also been implicated in fetal neurodevelopment, including processes such as neuronal migration, proliferation, differentiation, synapse function, and glia activation [19]. For example, chemokines, such as CXCR4, play an important role in progenitor cell migration and proliferation [44]. Tumor necrosis factor (TNFα) can mediate synaptic plasticity and strength during development, as well as receptor trafficking that impacts excitatory and inhibitory synapses [7, 63, 64]. Due to the dual role cytokines have in both immunity and CNS development, cytokine disturbances during gestation can alter neurodevelopment [19]. Increased maternal pro-inflammatory cytokines IFNy, IL-4, and IL-5 have been associated with a higher risk of having a child with ASD [25]. Moreover, Jones et al., found that mothers of children with ASD and comorbid intellectual disability have inflammatory profiles characterized by increases in GM-CSF, IL-6, IFNy, and IL-1α [35]. Individuals with ASD also had elevated levels of pro-inflammatory cytokines in the amniotic fluid [1]. Neonatal blood spot data also shows dysregulated cytokine levels in children later diagnosed with ASD [36, 38]. Maternal immune activation (MIA) models, whether induced via infectious agents or maternal conditions, have been associated with ASD-like behaviors in adult offspring and altered levels of cytokines in the brain [23, 54, 57, 65, 66].

In this study we set out to determine what consequences, if any, viral infection-based inflammation during pregnancy can have on the developing placenta and fetal brain in mice. We used multiplex cytokine analysis and bulk RNA sequencing of mouse fetal tissues to assess the impact of MIA on offspring neurodevelopment using the viral mimic poly I: C.

## Methods

### Animals

C57BL/6 mice were purchased from Taconic Biosciences and maintained at University of California, Davis at the UC Davis Sacramento campus, Sacramento, CA. Mice were housed in ventilated cages on a 12-hour light/dark cycle with same sex littermates at 23 °C. Food and water was provided *ad libitum.* All procedures were performed with approval by University of California Davis Institutional Animal Care and Use Committee and according to guidelines established by National Institute of Health Guide for the Care and Use of Laboratory Animals.

### Maternal immune activation

8–10 week-old female C57BL/6 mice were paired with males. Insemination was confirmed by the presence of a vaginal plug the following morning, marking the gestational time as embryonic day (ED) 0.5. On embryonic day 12.5, pregnant dams were intraperitoneally administered 20 mg/kg γ-irradiated poly I: C (Sigma) to stimulate maternal immune activation. Control groups were administered saline. On ED17.5, fetal brains and placenta were collected during the murine light cycle and flash frozen and stored at -80 until cytokine and RNA analysis.

### Tissue collection

5 days following the poly I: C injection on ED17.5, pregnant dams were euthanized using CO_2_. The uterine horn was dissected out and placenta and fetal brains were taken from each of the offspring and flash frozen with dry ice. Tissues were lysed and the total protein was measured using a Bradford assay. Biological sex of fetal samples was determined using PCR genotyping of the SRY gene. Total protein concentration was measured using a Bradford assay.

### Multiplex bead-based cytokine analysis

Cytokines in the lysed tissues were quantified using a multiplex mouse T_H_17 bead-based assay. To account for litter effect, 1 male and 1 female from a total of 8 litters per treatment group were used for cytokine analysis (i.e. *n* = 16 MIA offspring vs. *n* = 16 saline controls). The following cytokines were quantified: CD40 Ligand (CD40L), Granulocyte-macrophage colony stimulating factor (GM-CSF), Interferon gamma (IFN-γ), Interleukin 1-beta (IL-1β), IL-2, IL-4, IL-5, IL-6, IL-10, IL-12 (p70), IL-13, IL-15, IL-17 A, IL-17E/IL-25, IL-17 F, IL-21, IL-22, IL-23, IL-27, IL-28B, IL-31, IL-33, macrophage inflammatory protein 3-alpha (MIP-3α/CCL20), tumor necrosis factor alpha (TNF-α), and lymphotoxin (LT-α). Each tissue sample was diluted to a standardized concentration of 80 µg and run in duplicate. 25 ul of sample, standard, or quality control was loaded into each well and incubated overnight with antibody-coupled magnetic beads. After overnight incubation, the plate was washed using a Bio-Plex handheld magnet (Bio-Rad Laboratories, Hercules, CA, USA). The plate was then incubated for one hour with biotinylated detection antibody and 30 min with streptavidin-phycoerythrin before reading on a Bio-Rad Bio-Plex 200 plate reader (Bio-Rad Laboratories, Hercules, CA, USA). The minimum limit of detection is as follows: IL-17E/IL-25: 357.3 pg/mL; GM-CSF: 19.8 pg/mL; IFNγ: 1.5 pg/mL; MIP-3α:/CCL20: 9.4 pg/mL; IL-1 β: 3.3 pg/mL; IL-2: 1.5 pg/mL; IL-4: 0.4 pg/mL; IL-5: 5.1 pg/mL; IL-6: 3.3 pg/mL; IL-21: 35.8 pg/mL; IL-22: 0.5 pg/mL; IL-28B: 29.3 pg/mL; IL-10: 5.6 pg/mL; IL-23: 70.4 pg/mL; IL-12p70: 12.9 pg/mL; IL-27: 498.6 pg/mL; IL-13: 32.1 pg/mL; IL-15: 8.6 pg/mL; IL-17 A: 18.6 pg/mL; IL-17 F: 5.2 pg/mL; IL-33: 14.9 pg/mL; IL-31: 25.8 pg/mL; LT-α: 137.1 pg/mL; TNF-α: 1.3 pg/mL; CD40L: 14.6 pg/mL. Concentrations that fell below the minimum level of detection were given a value of half the limit of detection. Analytes where more than 60% of samples were above the level of detection were included in further statistical analysis. All fetal brain samples were in range, however, in the placenta, analytes below levels of detection and not included were as follows: IL-1β, IL-4, IL-5, IL-12p70, IL-13, IL-15, IL-17 A, IL-17 F, IL-21, IL-22, IL-25/IL-17E, and CD40L.

### Bulk RNA sequencing

RNA was isolated from fetal brain (n = 32; 16 males and 16 females, per treatment group) and placentas (n = 32; 16 males and 16 females, per treatment group) from 16 litter, per treatment groups using RNA/DNA Miniprep Plus Zymogen Kit (Cat#D7003). To ensure purity, RNA was further treated with DNAse1 for 15 minutes. RNA quantification was done using a Qubit. Chemical purity was determined using a Nanodrop, where the absorbance ratios fell between 1.6 and 2.1(260/280) and above 1.3 (260/230). cDNA libraries were prepared according to the Lexogen manual, using 500 ng of total input RNA per sample and 12 cycles of PCR amplification. 64 unique, dual index barcodes were used and samples were then pooled. Samples were exonuclease VII treated and submitted for QuantSeq 3’ mRNA single-end sequencing on the HiSeq4000 to generate 100 bp single-end reads.

### RNA-seq analysis

Raw sequencing reads were assessed for quality control using FastQC and multiqc (PreTrim_multiqc.html). Umi indexes were added using umi2index (Lexogen) and then trimmed to remove adapter contamination, polyA read through and low-quality reads using BBDuk. All samples were then re-assessed for quality using FastQC and multiqc (PostTrim_multiqc.html). Samples were aligned to the mouse genome (mm10) using STAR. Aligned bam files were then filtered for only unique UMIs using collapse_UMI_bam (Lexogen) (STARAlignment.html). Filtered bam files were sorted and indexed using samtools. Reads per gene were then counted using FeatureCounts at the gene id levels with -s 1 designated for stranded RNAseq. Counts were then read into R for statistical analysis using EdgeR and LimmaVoom. To remove low-expressing genes, only genes with greater than 1 count per million (CPM) reads in at least 16 of the total libraries were included in the analysis (Figure S1). The remaining 16,546 genes were then normalized using Trimmed Mean of M-values (TMM) to correct for library composition using calcNormFactors, method = TMM (Figure S2). The resulting CPM values were then fed into Voom using a design matrix with factors for treatment, sex and tissue (combined into one group variable) with uterine horn position as a covariate (∼0 + group + uterine.horn.position). Within subjects correlation for dam was removed by feeding the results of duplicateCorrelation into lmFit and including dam identification as a blocking factor. Individual contrast comparisons for each sex or collapsed across sex (brain saline vs. poly I: C, placenta saline vs. poly I: C, brain saline vs. placenta saline and brain poly I: C vs. placenta poly I: C) were then called using contrasts.fit followed by eBayes. The contribution of each factor to the overall gene expression profile was assessed using Multi-Dimensional Scaling plots (Figure S3-S5).

### Data availability

Fastq files and counts files for each sample are available on NCBI GEO at GSE248222 and analysis code is available on the Ciernia Lab github: https://github.com/ciernialab.

### Statistical analysis

For cytokine analysis, cytokine levels were assessed via Luminex and entered into Graphpad Prism (v 9.4.1) for statistical analysis. Outliers were identified using ROUT, with a Q = 1%. Outlier free data was used moving forward. Data was determined to be non-parametric. Prior to outlier removal, the n count in each treatment group was *n* = 8 males and *n* = 8 females. For data, stratified by sex, Kruskal-Wallis test with Dunn’s test was performed to account for multiple comparison testing, with a p-value of *p* < 0.05 as significant. All p-values were calculated to 4 decimal places. Data is represented as Median and IQR (25th -75th percentile). For RNA-Seq analysis, differentially expressed genes (DEG) were identified for each comparison of interest using the decideTests function with a Benjamini Hochberg (BH) correction and method = separate. Significant differentially expressed genes (BH corrected *p* < 0.05) between treatments were compared across tissues using VennDiagram. The web-based application Pathway, Network and Gene-set Enrichment Analysis (PANGEA) was used for gene ontology (GO) and Kytoo Encyclopedia of Genes and Genomes (KEGG) pathway analysis with a background of all genes in the analysis [30]. Sex was included as a variable in the analysis and results are reported both aggregated across sex and separated by sex (as discussed above). Each comparison was corrected for using Benjamini Hochberg to q < 0.05. The code for analyses is accessible at: https://github.com/ciernialab/Ashwood-MIA-RNAseq-2023/tree/main.

## Results

### MIA offspring have increased cytokines in the placenta

**Fig. 1:**
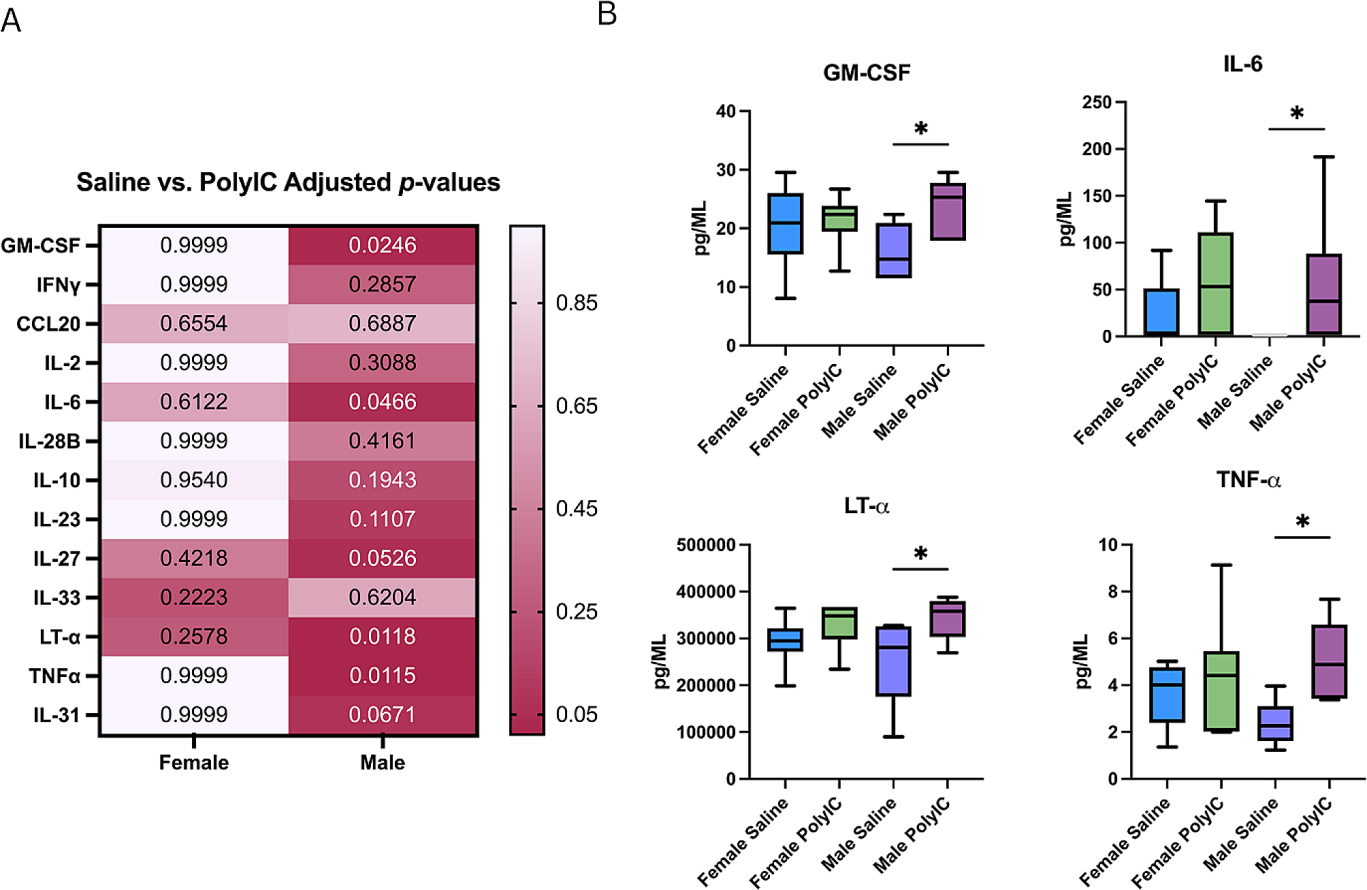
Male MIA offspring have increased cytokines in the placenta. (**A**) Heat map of Kruskal-Wallis p-values adjusted for multiple comparisons (Dunn’s test) of all cytokines analyzed within the range of detection in the placenta. Color intensity is varied based on adjusted p-value result. (**B**) Cytokines found to be significant when compared to saline controls in the placenta analyzed via Kruskal-Wallis test with Dunn’s multiple comparisons test. Significance was determined if adjusted *p* < 0.05. Data is shown as box and whisker plots with median and IQR (25th -75th percentiles), with tails at 5th and 95th percentiles. Significance is shown as * = *p* < 0.05; ** = *p* < 0.01

T_H_17 cytokines are typically produced as a response to extracellular infection and have important roles in brain development and behavior [15, 28, 45, 75]. Interestingly, T_H_17 related cytokines and the cells that produce them are essential for MIA induced behavioral changes, such as reduced social interactions and repetitive behavior, in rodent offspring [15]. Due to the data surrounding T_H_17 involvement in ASD, we chose to investigate T_H_17 related cytokine levels in MIA offspring. Placentas were collected at ED17.5 and analyzed for differences in T_H_17 cytokines. Male MIA offspring showed significant increases in GM-CSF (*saline median = 14.76 pg/mL; poly I: C median = 25.30 pg/mL; p = 0.0246*), IL-6 (*saline median = 1.65 pg/mL; poly I: C median = 47.35 pg/mL; p = 0.0466)*, LT-α (*saline median = 280,444 pg/mL; poly I: C median = 357,924 pg/mL; p = 0.0118*) and TNFα (*saline median = 2.27 pg/mL; poly I: C = 4.89 pg/mL; p = 0.0115;*) (Fig. 1a and b; Table 1.) Female offspring showed no significant difference in placental cytokines compared to the control group (Fig. 1b; Table 2). Together, these data demonstrate that exposure to poly I: C during pregnancy can cause an increase in placental cytokines stable after 5 days and this phenomenon is sex dependent.

**Table 1:** Statistics of male placenta cytokines. Data is represented as Median and IQR (25th -75th percentiles)

Cytokine	Saline	Poly I: C	*P*-value
IFNγ	7.75 (0.75–22.93)	17.44 (8.94–29.80)	0.41
CCL20	4.70 (4.70–4.70)	4.92 (4.70-19.71)	0.34
IL-2	5.30 (1.24–7.57)	8.23 (4.31–9.80)	0.18
IL-6	1.65 (1.65–1.65)	47.35 (8.55–165.8)	0.04*
IL-10	2.80 (2.80–5.69)	7.44 (3.39–22.05)	0.14
IL-23	35.20 (35.20–35.20)	297.9 (35.20–17,823)	0.08
IL-27	510.1 (138.3-555.5)	804.9 (491.8–825.0)	0.11
IL-28B	80.56 (49.95–107.7)	104.7 (78.66–134.0)	0.27
IL-31	59.07 (12.9-212.9)	382.0 (127.8-456.7)	*0.07*
IL-33	7.45 (4.09–7.45)	8.18 (2.46–12.81)	0.27
TNFα	2.27 (1.61–3.10)	4.89 (3.43–6.59)	0.01*
LT-α	280,444 (175,445 − 326,094)	357,924 (303,291–379,754)	0.01*
GM-CSF	14.76 (11.49–20.91)	25.30 (17.89–27.79)	0.02*

**Table 2:** Statistics of female placenta cytokines. Data is represented as Median and IQR (25th -75th percentiles)

Cytokine	Saline	Poly IC	*P*-value
IFNγ	12.70 (6.18–23.78)	9.16 (5.19–26.05)	0.98
CCL20	4.70 (4.70–4.70)	4.70 (4.70-20.79)	0.44
IL-2	6.12 (3.12–8.36)	5.82 (3.75–10.94)	0.76
IL-6	1.65 (1.65–51.41)	53.30 (1.65–111.1)	0.32
IL-10	2.80 (2.80–9.19)	3.39 (2.80-16.32)	0.41
IL-23	2,832 (35.20-8,851)	4,267 (35.20–17,048)	0.85
IL-27	532.7 (459.4-583.2)	667.9 (448.2-725.9)	0.08
IL-28B	100.2 (72.18–132.2)	98.67 (92.65–136.3)	0.84
IL-31	129.0 (27.48–229.4)	136.8 (12.90-352.4)	0.71
IL-33	2.41 (0.29–7.45)	7.98 (1.80-14.09)	0.17
TNFα	4.00 (2.39–4.76)	4.41 (2.02–5.46)	0.76
LT-α	294,768 (271,323 − 322,030)	348,105 (298,066–367,092)	0.09
GM-CSF	20.91 (15.54–26.01)	22.39 (19.41–23.85)	0.80

### MIA offspring have increased cytokines in the fetal brain

**Fig. 2:**
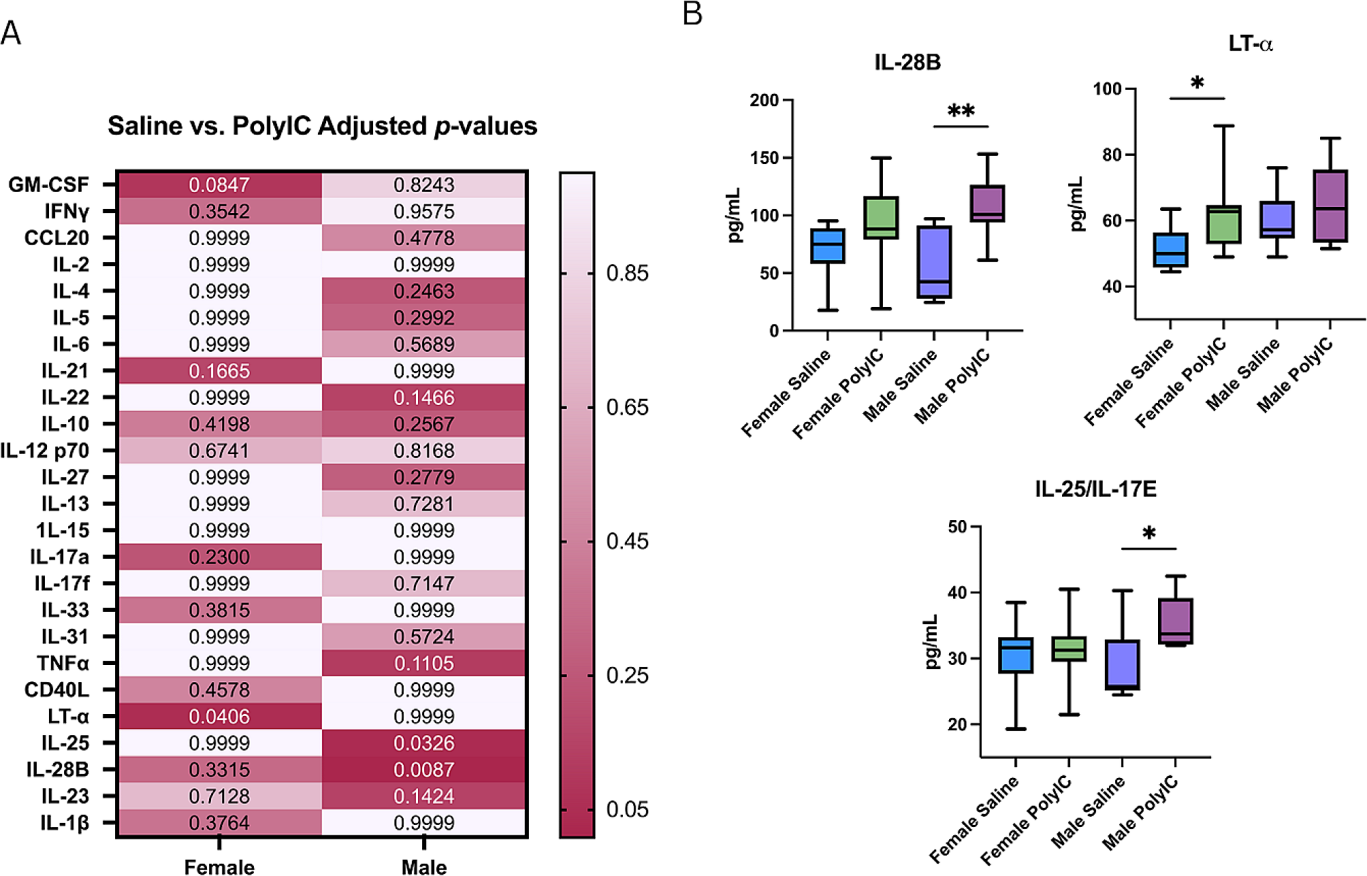
Cytokine increase in MIA fetal brains is sex-dependent. (**A**) Heat map of Kruskal-Wallis p-values adjusted for multiple comparisons (Dunn’s test) of all cytokines analyzed within the range of detection in the fetal brain. Color intensity is varied based on adjusted p-value result. (**B**) Cytokines found to be significant when compared to saline controls in the fetal brain analyzed via Kruskal-Wallis test with Dunn’s multiple comparisons test. Significance was determined if adjusted *p* < 0.05. Data is shown as box and whisker plots with median and IQR (25th -75th percentiles), with tails at 5th and 95th percentiles. Significance is shown as * = *p* < 0.05; ** = *p* < 0.01

Due to behavioral abnormalities seen in MIA offspring and their relationship with neuroinflammation, we assessed the cytokine content of MIA offspring fetal brains [15, 27, 31, 32]. Sex-dependent changes were seen in the offspring brains. In males, IL-28B (*saline median = 42.55 pg/mL; poly I: C median = 100.9 pg/mL; p = 0.0087)*, and IL-25 (*saline median = 25.75 pg/mL; poly I: C median = 33.75 pg/mL; p = 0.0326)* were significantly increased in MIA offspring (Fig. 2a and b; Table 3). LT-α was increased in the MIA female offspring only (*saline median = 50.0 pg/mL; poly I: C median = 62.75 pg/mL; p = 0.0406)* (Fig. 2a and b; Table 4). These results suggest that immune activation during pregnancy can affect the levels of proinflammatory cytokines in offspring brains and that there is sex dependent response.

**Table 3:** Statistics of male brain cytokines. Data is represented as Median and IQR (25th -75th percentiles)

Cytokine	Saline	Poly IC	*P*-value
IFNy	14.15 (13.08–16.38)	15.15 (13.45–17.58)	0.48
CCL20	12.15 (11.5-13.73)	12.9 (12.13–15.08)	0.29
IL-1β	20.4 (19.25–21.5)	20.9 (19.63–21.5	0.69
IL-2	27.25 (22.63–30.38)	27.25 (22.63–30.38)	0.90
IL-4	16.0 (14.0-16.5)	16.65 (16.0-17.83)	0.14
IL-5	10.25 (10.0-10.95)	11.25 (10.25–11.95)	0.10
IL-6	15.5 (14.33-16.0)	15.65 (14.98–17.95)	0.36
IL-10	20.15 (15.93-21.0)	24.15 (19.1–64.2)	0.22
IL-12p70	34.4 (32.1-32.38)	37.55 (35.08–41.13)	0.29
IL-13	15.4 (14.5-17.63)	16.8 (15.55–18.13)	0.34
IL-15	22.0 (20.98–25.88)	23.25 (21.25–25.48)	0.87
IL-17a	10.5 (10.0-11.23)	10.5 (10.13–10.88)	0.99
IL-17 F	31.4 (29.98–32.88)	33.4 (28.5-37.38)	0.42
IL-21	306.7 (296.7-328.9)	297.9 (262.1-319.3)	0.50
IL-22	16.25 (15.13–16.88)	18.3 (15.63–21.83)	0.14
IL-23	15.75 (15.0-17.83)	18.4 (16.5–20.6)	0.06
IL-25/IL17E	25.75 (25.13–32.88)	33.75 (32.13–39.18)	0.03*
IL-27	29.65 (22.5-32.38)	32.5 (25.38–36.48)	0.19
IL-28B	42.55 (27.98–91.18)	100.9 (93.88–126.5)	< 0.01**
IL-31	24.0 (18.55–26.2)	25.4 (19.75–28.5)	0.29
IL-33	33.5 (30.45–36.13)	34.9 (31.38–37.25)	0.52
TNFα	11.65 (11.13–12.83)	12.9 (11.85–13.88)	0.08
LTα	57.25 (54.63–65.95)	63.65 (53.35–75.48)	0.78
GM-CSF	15.4 (15.0-16.38)	16.25 (14.88–16.5)	0.35
CD40L	26.9 (23.88–29.5)	27.25 (26.13–28.23)	0.86

**Table 4:** Statistics of female brain cytokines. Data is represented as Median and IQR (25th -75th percentiles)

Cytokine	Saline	Poly IC	*P*-value
IFNγ	15.65 (13.0-16.23)	16.5 (14.0-17.38)	0.15
CCL20	12.75 (10.6–14.1)	12.15 (12.0–14.0)	0.94
IL-1β	20.5 (19.63–20.88)	20.9 (20.5-21.75)	0.13
IL-2	25.9 (23.88–28.98)	26.9 (22.73-28.0)	0.88
IL-4	14.8 (13.0-17.63)	15.5 (15.0-16.88)	0.37
IL-5	10.0 (9.78–11.43)	10.5 (9.5-11.38)	0.93
IL-6	16.25 (14.13–16.5)	15.5 (14.63–17.63)	0.81
IL-10	17.5 (14.88–23.08)	25.15 (17.7–33.1)	0.27
IL-12p70	37.75 (32.48–38.88)	39.0 (33.98–42.75)	0.37
IL-13	16.4 (13.63–18.25)	16.55 (15.0-17.5)	0.70
IL-15	25.25 (20.25–27.25)	24.4 (21.5-26.38)	0.94
IL-17 A	11.1 (11.0-11.73)	10.65 (9.93–11.75)	0.22
IL-17 F	32.95 (29.63–36.38)	32.65 (29.38–35.88)	0.88
IL-21	280.4 (250.1-314.3)	315.5 (291.6-332.7)	0.16
IL-22	17.5 (14.88–23.08)	16.75 (15.48–18.55)	0.94
IL-23	18.5 (15.28–19.88)	16.05 (14.63–18.88)	0.50
IL-25/IL17E	31.65 (27.7-33.25)	31.25 (29.5-33.38)	0.93
IL-27	32.4 (25.63–34.43)	27.65 (22.98–33.98)	0.59
IL-28B	75.0 (58.13–88.98)	88.25 (79.0-116.7)	0.11
IL-31	25.15 (20.98–25.88)	22.55 (19.33–26.73)	> 0.99
IL-33	32.75 (30.5-33.88)	34.25 (32.63–38.6)	0.20
TNFα	13.0 (12.5-13.45)	12.75 (11.85–14.13)	0.59
LTα	50.0 (45.75–56.38)	62.75 (52.88–64.68)	0.02*
GM-CSF	16.9 (15.63–18.18)	15.15 (14.5-16.88)	0.0777
CD40L	27.1 (25.38–27.73)	28.0 (26.75–29.05)	0.1657

### MIA offspring placenta have DEG enriched in biological processes essential for neurodevelopment

**Fig. 3:**
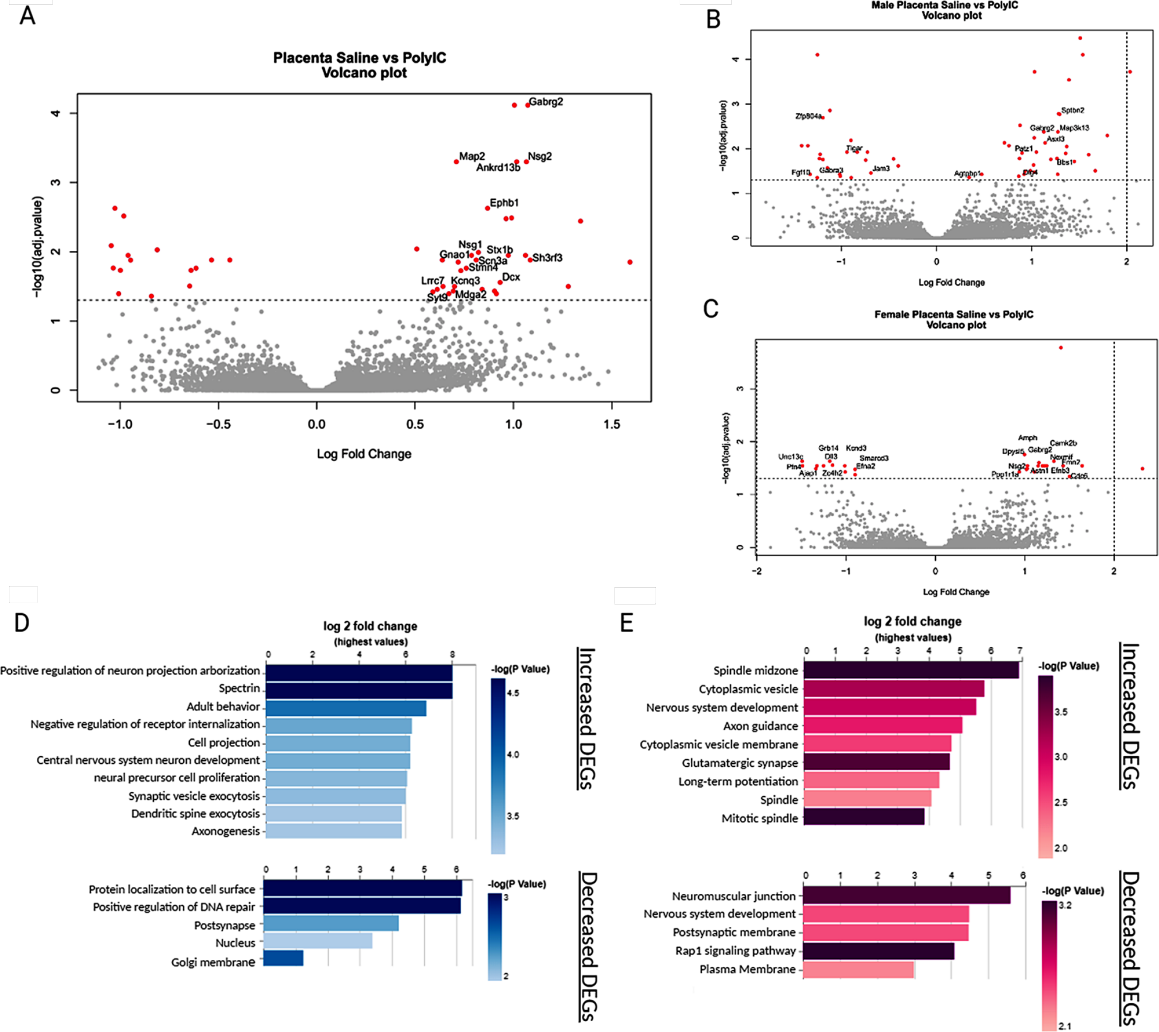
Differentially expressed genes and their biological pathways in the placentas of male and female MIA offspring. (**A** Volcano plot of placental DEG from MIA and saline offspring. Genes were considered differentially expressed when the Benjamini Hochberg (BH) corrected p-value < 0.05 between conditions. Male (**B**) and female (**C**) DEG between MIA and saline groups are represented using volcano plots. Significantly enriched GO and KEGG terms in male (**D**) and female (**E**) placental DEG are displayed. Significantly enriched terms were determined significant if BH adjusted p-values were less than *p* < 0.05

Our analysis of the placenta from MIA offspring revealed a total of 47 DEG, with 15 decreased and 32 increased in MIA 5 days following poly I: C injection on E17.5 compared to saline samples (Fig. 3a). Upon comparing enrichment terms between increased and decreased genes, distinct GO terms emerged. Among increased DEG, many of the significantly enriched terms were related to neuronal processes, such as ‘neuron projection’, ‘dopamine receptor signaling pathway’ and ‘axon initial segment’ (Supplemental File 2), all of which were enriched in genes that regulate synaptic vesicle trafficking (neuronal vesicle trafficking associated 2; *Nsg1* and *Nsg2*) and cytoskeletal movement (*Ephb1* and *Stmn4*). The term ‘dopamine receptor signaling pathway‘ was also enriched, alongside DEG associated with excitatory and inhibitory neurotransmitter activity (*Gabrg2*, *Lrrc7*, and *Gnao1*). After correction for multiple testing, no significantly enriched GO term or KEGG pathway was found among the decreased DEG, though the GO term ‘positive regulation of synapse assembly’ encompassing genes such as cut-like homeobox 2 (*Cux2*), Slit and NTRK-like family member 4 (*Slitrk4*) and adhesion G-protein-coupled receptor L1 (*Adgrl1*) trended towards significance (adjusted p-value = 0.09) (Supplementary File 2). When the data was stratified by sex, 56 placental DEG (34 increased, 22 decreased) were found between male MIA and male saline offspring placenta and 28 placental DEG (17 increased, 11 decreased) between female MIA and female saline offspring placenta (Fig. 3b and c). Increased male DEG were related to nervous system development (‘central nervous system neuron development’, ‘neural precursor cell proliferation’ and ‘cell projection’), which all consisted of genes involved in synaptic communication (Discs large MAGUK Scaffold Protein 4; *Dlg4*, *Nsg2*) and the cytoskeleton (Spectrin beta, non-erythrocytic 2; *Sptbn2*, FYVE, RhoGEG and ph domain containing 2;*Fyve*, microtubule associated protein 2; *Map2*). Decreased DEG in male MIA offspring were enriched in the GO Biological Process terms related to protein localization (‘protein localization to cell surface’) and DNA repair (‘positive regulation of DNA repair’), which included genes involved in tissue growth and repair (fibroblast growth factor 10; *Fgf10*, and junctional adhesion molecule 3; *Jam3*) (Fig. 3d). In female offspring, GO and KEGG terms from increased DEG were related to neurotransmitter signaling and synaptic vesicles (‘glutamatergic synapse’, ‘Long-term potentiation’, ‘Adrenergic signaling in cardiomyocytes’, and ‘cytoplasmic vesicle membrane’), as well as neuronal development and connectivity (‘nervous system development’ and ‘Axon guidance’) (Fig. 3e). Decreased female DEG were also associated with cell membranes and synapses (Fig. 3e). In summary, these results suggest that offspring exposed to MIA during gestation have disrupted neuronal development that is observable in the placenta.

### MIA offspring fetal brain have DEG enriched in biological processes essential for neurodevelopment

**Fig. 4:**
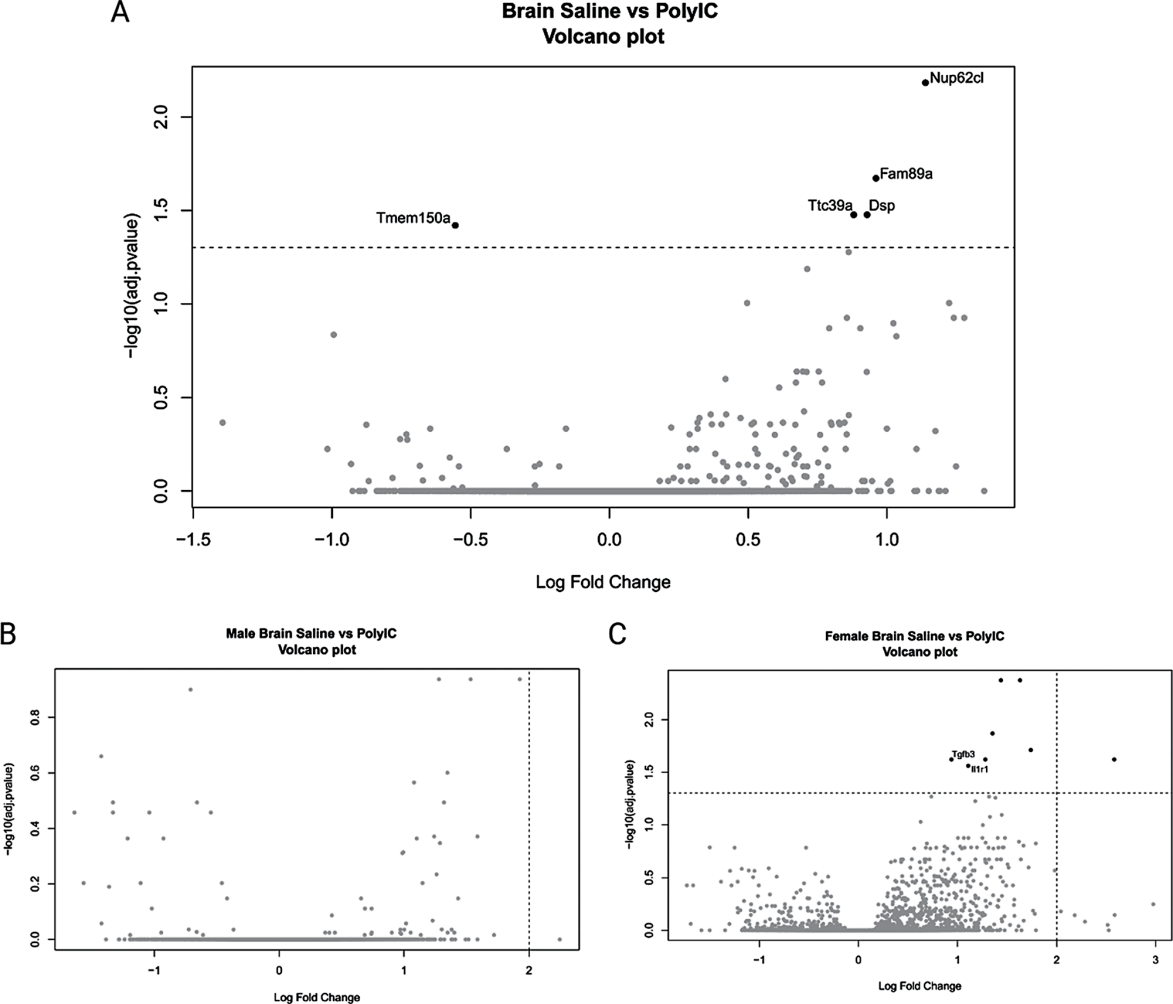
Differentially expressed genes in the fetal brains of MIA offspring. Volcano plot of DEG found in the fetal brain of MIA offspring compared to saline offspring. Male (**B**) and female (**C**) data is represented using volcano plots. Genes were considered differentially expressed when the BH corrected p-value *p* < 0.05 between conditions

Next, we assessed the impact of MIA on the developing fetal brain transcriptome. In response to poly I: C treatment, after 5 days, we identified 5 DEG, with 4 increased and 1 decreased in expression for MIA relative to saline (Fig. 4). Moreover, few sex effects were observed between treatment groups (8 increased DEG in female MIA vs. saline offspring, but none in male MIA vs. saline offspring) (Fig. 3b and c). While some significant GO terms were identified in female MIA offspring (‘Amoebiasis’, ‘Cytokine-cytokine receptor interaction’ and ‘Human T-cell leukemia virus 1 infection’), all were driven by the genes interleukin 1 receptor type 1 (*Il1r1*) and transforming growth factor beta 3 (*Tgfb3*) (Fig. 3c). To summarize, MIA fetal brains 5 days post poly I: C treatment mostly impact female offspring and genes involved in the immune response.

## Discussion

**Fig. 5:**
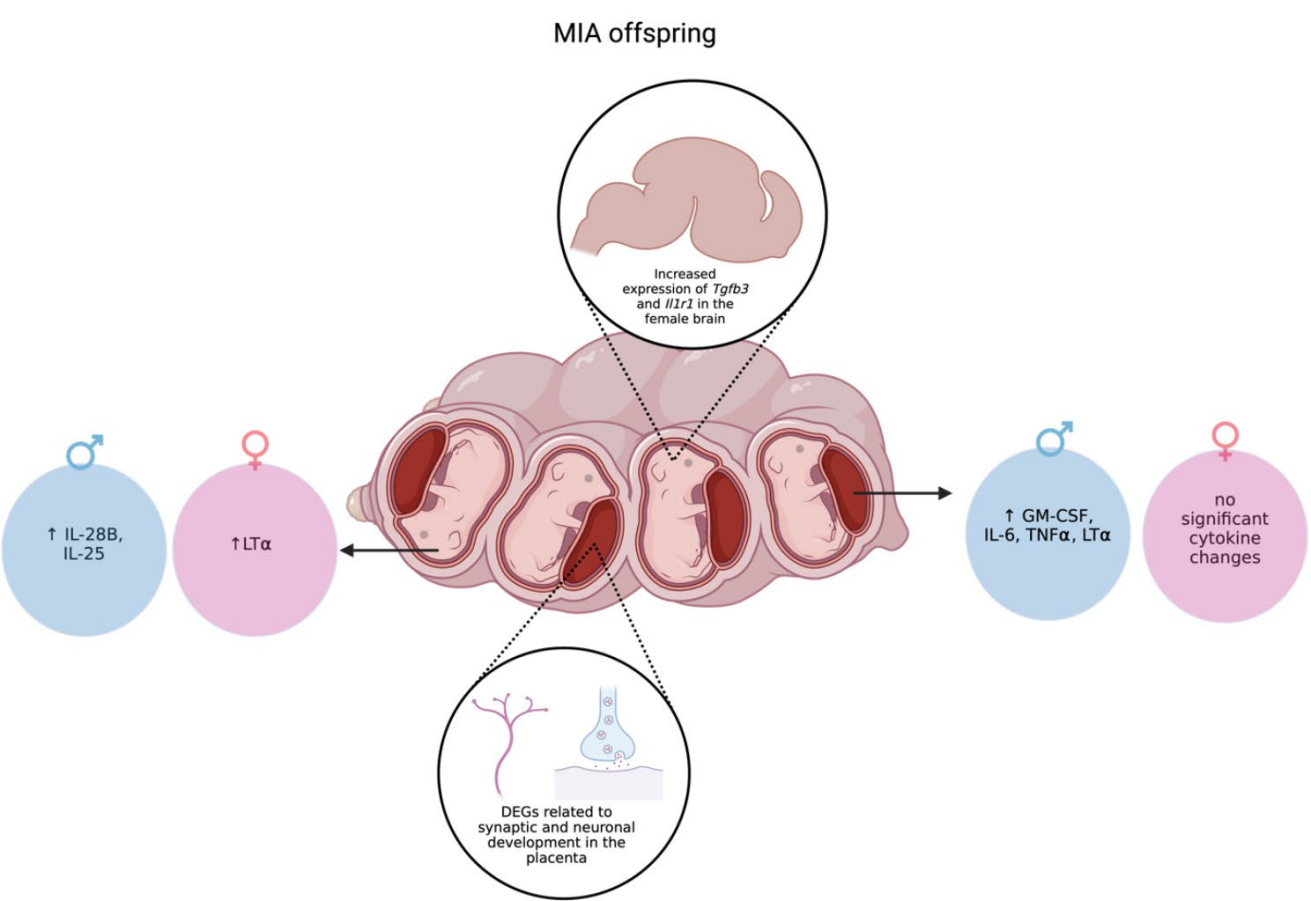
Summary of Cytokine and Gene Expression Changes in MIA Offspring. Made with biorender.com

The maternal immune environment during pregnancy is an important factor in fetal brain development. Inflammation during pregnancy can lead to alterations in behavior and neurodevelopment in the offspring. We hypothesized that we would see changes in cytokine levels and gene expression in offspring of dams exposed to the viral mimic poly I: C during pregnancy. Due to the high prevalence of ASD in males as compared to females, we also expected to see sex dependent effects on fetal brain and placenta cytokine levels and transcriptomics. Indeed, we saw increases in cytokines in both the placenta and brains of male MIA offspring, and no significant changes in the female MIA group. We also observed altered gene expression in the placenta and fetal brain of MIA offspring and with a sex dependent effect seen in the mRNA data between male treatment groups. Overall, our findings show that MIA causes changes in the fetal environment that impact neuroinflammation and neurodevelopment at 5 days post inflammatory event (Summary Fig. 5).

Fetal programming is the process by which environmental stress and epigenetic changes during pregnancy alter fetal organ development. This concept suggests that altered maternal health, including immune function, can result in more adverse health outcomes for the offspring, with the placenta playing an essential role [12, 39]. During pregnancy, the placenta relays signals from the mother to the developing fetus and acts to supply the fetal organs, especially the brain, with necessary nutrients [11]. As a result, the placenta can participate in the transfer of inflammatory cytokines from the maternal circulation to the developing fetus, though this method of transfer appears dependent on the type of cytokine studied. In an MIA model with pregnant rats, radiolabeled maternal IL-6 crossed placental tissues into fetal circulation during mid-gestation, whereas IL-1β transfer was minimal [17, 26]. Moreover, the placenta is a significant endocrine organ itself and is capable of producing its own inflammatory mediators upon exposure to inflammation [29, 37, 76]. Another possibility for increased placental inflammation may be that it is reflecting the immune signature being delivered to the fetus and/or the fetal immune response itself. In male MIA offspring, we observe increased placental GM-CSF, TNFα, LT-α, and IL-6 and increased IL-28 and IL-25 in the brain. It is possible that there is a relationship between these tissues as there tended to be more elevated placental cytokines in the MIA group, whereas there are fewer increased cytokines in the fetal brain. This suggests that placental transfer of inflammatory cytokines to the fetus may occur but limited to certain cytokines. Further studies need to be done in order to show a correlation between inflammation in the placenta and fetal brain.

In the placenta of MIA male offspring, we saw significant increases in the pro-inflammatory cytokines IL-6, TNFα, and LT-α as well as the myeloid growth and differentiation factor GM-CSF. IL-6, a pro-inflammatory cytokine, can cross the placental barrier and is necessary to elicit the neurodevelopmental and behavioral effects seen in MIA offspring [76, 79]. Along with IL-6, TNFα, can also alter synaptic plasticity and inhibit long term potentiation in the hippocampus [9, 62, 67]. TNFα mediates inflammatory processes during infection or autoimmunity. In the brain, TNFα regulates synaptic plasticity and long-term potentiation in the hippocampus, and in cases of disease, can be neurotoxic by inducing neuronal dendritic loss in the brain [63, 78]. GM-CSF has functions that range from increasing cellular proliferation to perpetuating inflammatory immune responses and is found to be increased in the brains of ASD patients [41, 73].

Our fetal brain data show sex-dependent increases in IL-25, IL-28B and LT-α. IL-28B is an interferon with antiviral properties [74]. Little is known about a possible function of IL-28B in neurodevelopment or neuroinflammation. IL-25, also known as IL-17E, can have both pro-inflammatory and anti-inflammatory effects [18]. Despite being part of the IL-17 family, IL-25 acts more similarly to a T_H_2 cytokine, inducing IL-4, IL-5, and IL-13 expression [50]. Increases in T_H_2 cytokines are seen in ASD and have previously been associated with improved cognitive outcomes [3, 4, 49]. LT-α, formerly known as TNFβ, was significantly increased in male placentas and female fetal brains of MIA offspring. LT-α is a pro-inflammatory, cytotoxic cytokine secreted by lymphocytes and can be secreted by microglia [10, 42]. LT-α has been shown to be involved in regulating cerebral microvasculature and inflammatory processes in the CNS [33, 56, 71]. LT-α induces mRNA expression of adhesion molecules ICAM-1, VCAM-1, P-selectin and E-selectin in brain endothelial cells and this expression is further increased with IFNy [71]. Elevated LT-α concentration can therefore have important implications for fetal brain development, specifically in neurodevelopmental events that require adhesion, such as cortical lamination events. Unexpectedly, we do not find this reflected in the sequencing analysis, with the exception of increased *Tgfb3* in the female MIA brains. *Tgfb3*, which encodes for the cytokine TGFβ3, and can induce cell differentiation and adhesion and may contribute to development itself [47]. It is likely that dysregulated TGFβ3 may be a part of a larger cytokine dysregulation within the female fetal brain, as increased *Il1r1* was also observed, and encodes for a receptor involved in IL-1α mediated pro-inflammatory reactions. These results support the idea that adhesion molecules may be dysregulated in ASD as a consequence of inflammation. Dysregulated adhesion molecules have long been documented in the context of ASD. Increased expression in adhesion gene molecules may be related to findings of increased cortical lamination in a similar MIA model at 5 days post poly I: C injection at ED17.5 in MIA offspring [13]. It is hypothesized that differences in brain development within the MIA model may result from changes in alterations within the neural progenitor cell (NPC) compartment [69]. Long-lasting neurodevelopmental changes driven by *in utero* exposure to inflammatory cytokines appear to stem from changes in neural stem cell populations [21]. It is possible that exposure to inflammatory cytokines changes the adhesive properties of NPC and other cells within the cortex, disrupting normal cortical lamination events. Abnormal cortical layering in patients with tuberous sclerosis complex (TSC) mutations, also a syndromic form of ASD, are observed, alongside increased expression of inflammatory and adhesion related genes [8]. Moreover, genome-wide association studies have revealed associations between single-nucleotide polymorphisms in cadherin 9 and cadherin 10 genes and ASD [70]. In the plasma of children with ASD, levels of soluble PECAM and P-selectin were found to be lower than in typically developing children [52]. The disrupted expression of adhesion molecules during brain development can potentially lead to abnormal neuronal migration and altered connectivity. Consequently, neuronal cells and their ability to form synaptic/puncta adherent junctions with other cells may be negatively impacted [68].

Among the upregulated placental DEG, pathway analysis showed that these were involved in many terms related to synaptic vesicles and neurodevelopment. Several genes implicated in excitatory and inhibitory signaling (*Gabrg2*, *Gnao1*, and *Kcnq3*), synaptic vesicle-mediated transport (*Syt9 and Stx1b)*and receptor recycling(*Nsg1* and *Ankrd13b*) were upregulated. In addition to synaptic dysregulation, the KEGG pathway analysis of increased placental DEG in MIA offspring, revealed an enrichment of genes involved in neuronal structures such as axons and dendrites, and genes involved in regulating cytoskeletal activity (*Map2*, *Pfn4*, *Stmn4*, and *Dcx*) and axonal growth (*Dpys15* and *Slitrk4*) in both sexes. Several of the MIA increased placental genes were also associated with various CNS disorders, such as epilepsy (*Gnao1* and *Scn3a*). It is well understood that placental production of neurotransmitters, such as serotonin and a variety of catecholamines, aids in fetal neurodevelopment [55]. However, the expression of nervous system related genes in the placenta and how that relates to development in gestation is poorly understood. In a mouse model exposed to polycholorinated biphenyl during gestation, increased demethylation in synapse and cell adhesion genes were noted in the placenta as well as altered neurodevelopment [40]. These data suggest that there may be a role for CNS gene expression in placenta during neurodevelopment. Placental development is dependent on its adherence to the uterine wall and is in part mediated by syncytiotrophoblasts (SCT). Recently, SCT were identified to be enriched in genes related to neuronal projection, axons and synapses [48]. Thus, it is possible for gene programs related to nervous system processes, specifically those that regulate adherence and synapses, to also be involved in placental function. *Fgf10 and Jam3*, two genes that were differentially expressed in the placenta of male offspring, are shown to be involved in trophoblast function and placenta development. The FGF10 protein is involved in trophoblast invasion and migration, making it important in formation of the placenta [51, 77]. *Jam3* expression was shown to be important in trophoblast fusion, as well as differentiation [14]. Some DEG found in the MIA placenta have previously been shown to alter placental development, but many placental DEG may also be involved in altered fetal neurodevelopment. In the current study, the placentas of male and female MIA offspring had increased expression of genes related to cytoskeletal and synaptic activity, which may be reflective of dysregulated placental development in MIA offspring. Furthermore, female offspring were also enriched in terms related to excitatory signaling. Others have found that gestational MIA alters the switch between excitatory and inhibitory signaling in offspring, potentially driven by IL-1R1 activity [16]. Altered placental production of allopregnanolone in females, a hormone that regulates gamma-aminobutyric acid receptors and therefore the excitatory/inhibitory balance, also contributes to altered behavior [6]. Therefore, it is possible that gestational MIA results in a female specific effect on the placental programs involved in the excitatory and inhibitory systems.

This study was limited by the inability to predict behaviors associated with the fetal tissue measurements. Although we have seen altered behavior in previous studies using poly I: C MIA, this current study had no behavioral data. This study also did not have assays that measured maternal serum cytokine data so any maternal cytokine levels that may have impacted the fetal tissue cytokine levels and gene expression are unknown. The timing of the study is also limited in that tissues were taken 5 days post-injection, so any immediate inflammatory changes prior to this time are unknown and warrant further investigation. Our study results may therefore vary from other MIA studies due to this timeline to look at stable changes in DEG and cytokines after 5 days rather than immediate responses (within 2–3 h) post a poly I: C exposure.

## Conclusion

Maternal inflammation during pregnancy is frequently regarded as a causative factor of altered neurodevelopment and subsequent NDD diagnosis. Our data show a distinct profile of cytokines and gene expression in the mouse placenta and fetal brain of MIA offspring that arises as a result of mid-gestational poly I: C exposure. We also observed an increased sex-dependent cytokine response in the placenta and fetal brains of male MIA offspring, further supporting the notion that male offspring are more susceptible to gestational perturbations. Our findings demonstrate that MIA offspring exhibit dysregulated genes related to synaptic vesicles, neuronal development, adhesion and metabolic processes in fetal brains and placenta, which may contribute to the observed structural and neuronal connectivity issues frequently found in NDD.

### Electronic supplementary material

Below is the link to the electronic supplementary material.

Supplementary Material 1

Supplementary Material 2

Supplementary Material 3

Supplementary Material 4

## Data Availability

Availability of data and materials: The datasets used and/or analyzed during the current study are available from the corresponding author on reasonable request.Fastq files and counts files for each sample are available on NCBI GEO at GSE248222 and analysis code is available on the Ciernia Lab github: https://github.com/ciernialab.

## Abbreviations

ASD: Autism spectrum disorder
MIA: Maternal immune activation
poly (I: C): Polyinosinic: polycytidylic acid
IL: interleukin
DEG: differentially expressed genes
NDD: neurodevelopmental disorder
LPS: lipopolysaccharide
TNF: Tumor necrosis factor
CNS: central nervous system
IFN: interferon
ED: embryonic day
GM-CSF: granulocyte-macrophage colony stimulating factor
MIP: macrophage inflammatory protein
LT: lymphotoxin
CPM: counts per million
TMM: trimmed mean of M-values
BH: Benjamini Hochber
PANGEA: pathway network and gene-set enrichment analysis
GO: gene ontology
KEGG: Kytoo encyclopedia of genes and genomes
