# Supplementary material for: Impact of maternal immune activation and sex on placental and fetal brain cytokine and gene expression profiles in a preclinical model of neurodevelopmental disorders
Source: J Neuroinflammation. 2024 May 7;21:118. doi: 10.1186/s12974-024-03106-7 (PMC11077729; doi:10.1186/s12974-024-03106-7)

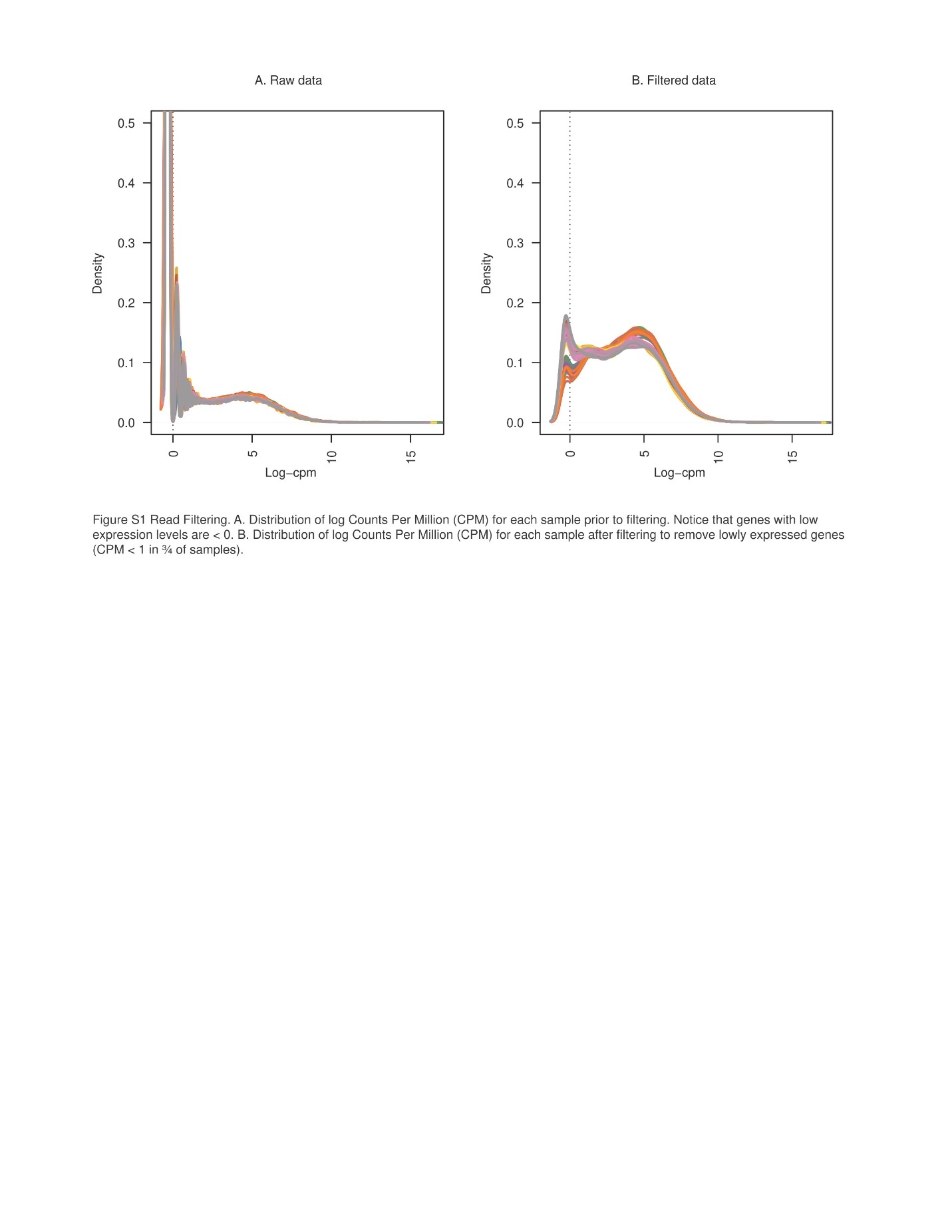


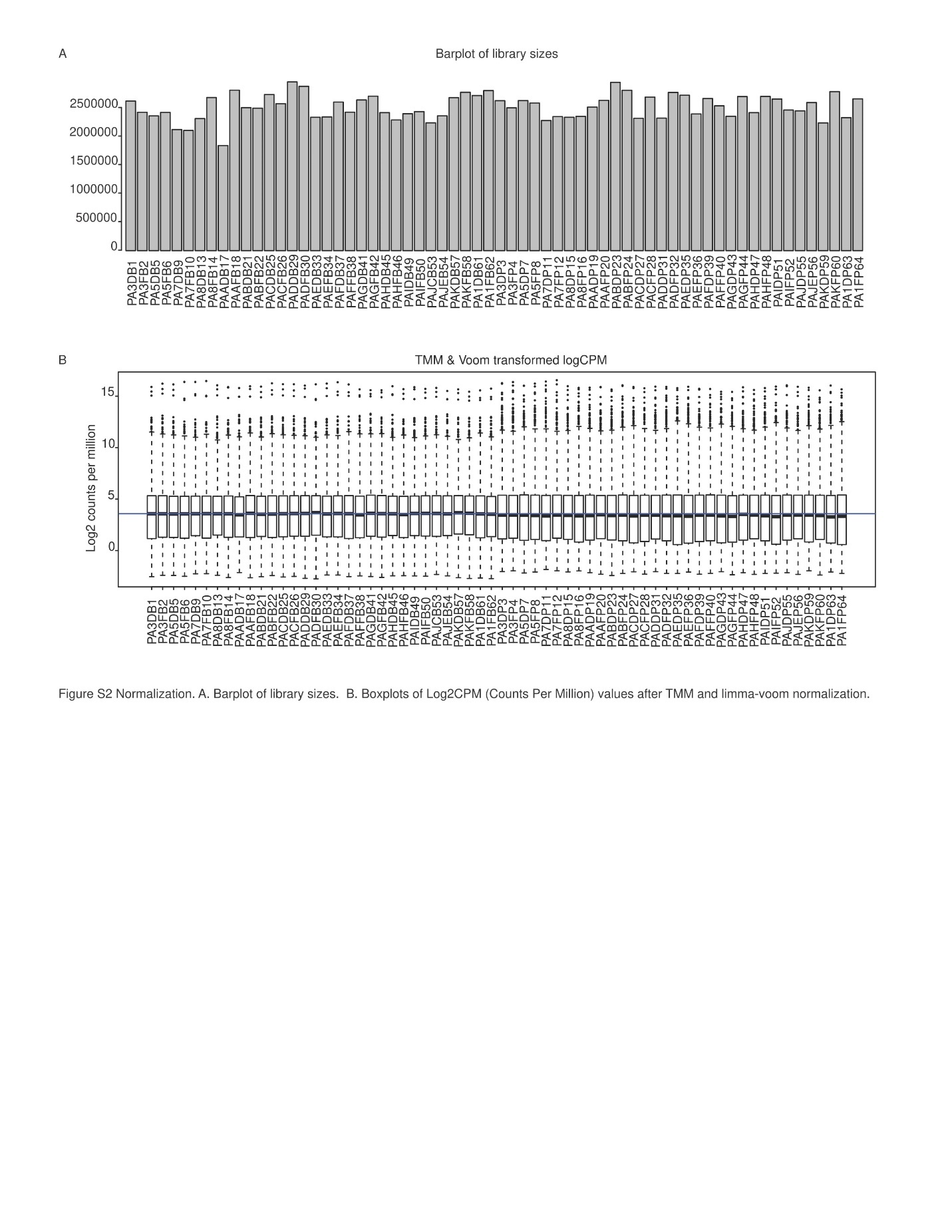


Figure S2 Normalization. A. Barplot of library sizes (read depth). B. Boxplots of Log2CPM (counts per million) values after TMM and limma-voom normalization.


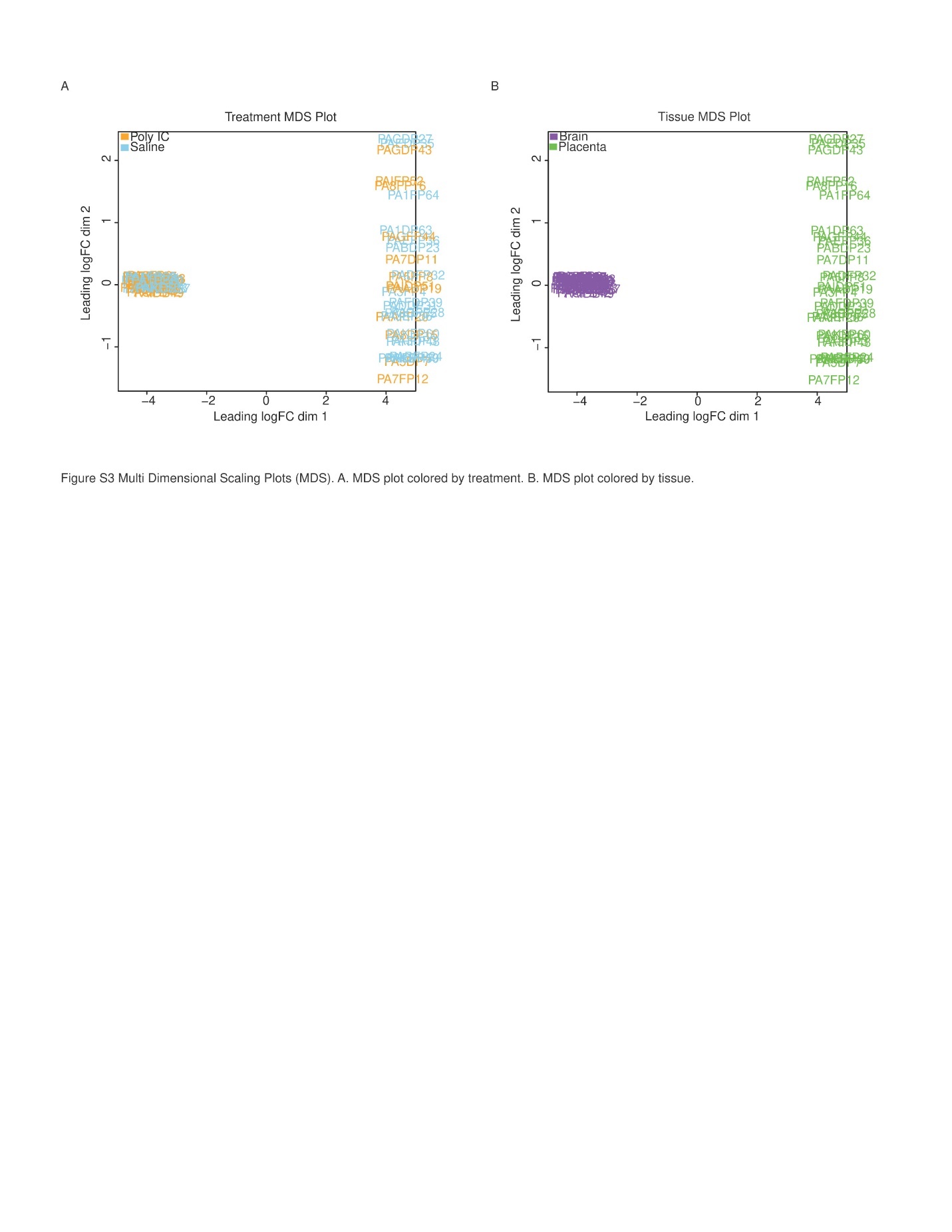

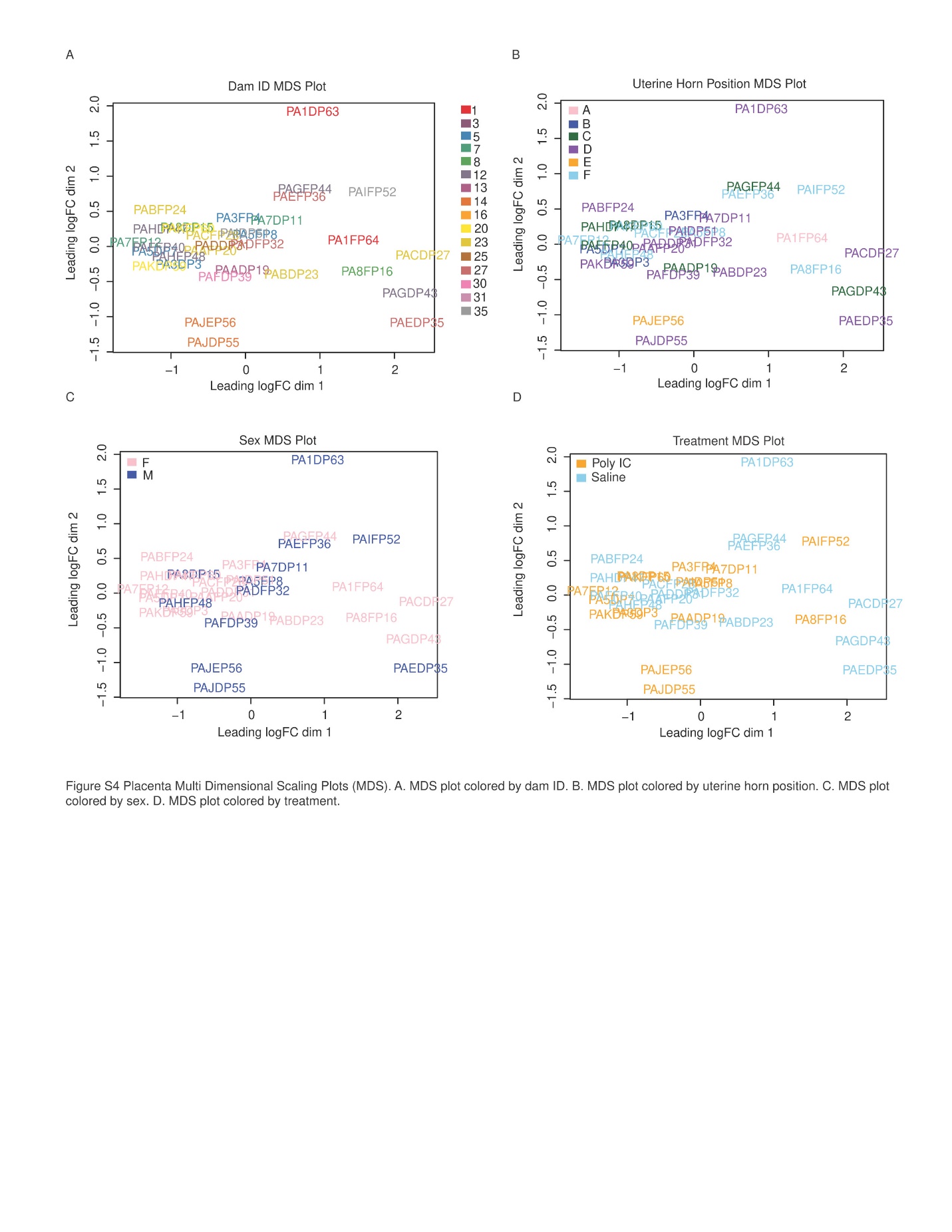


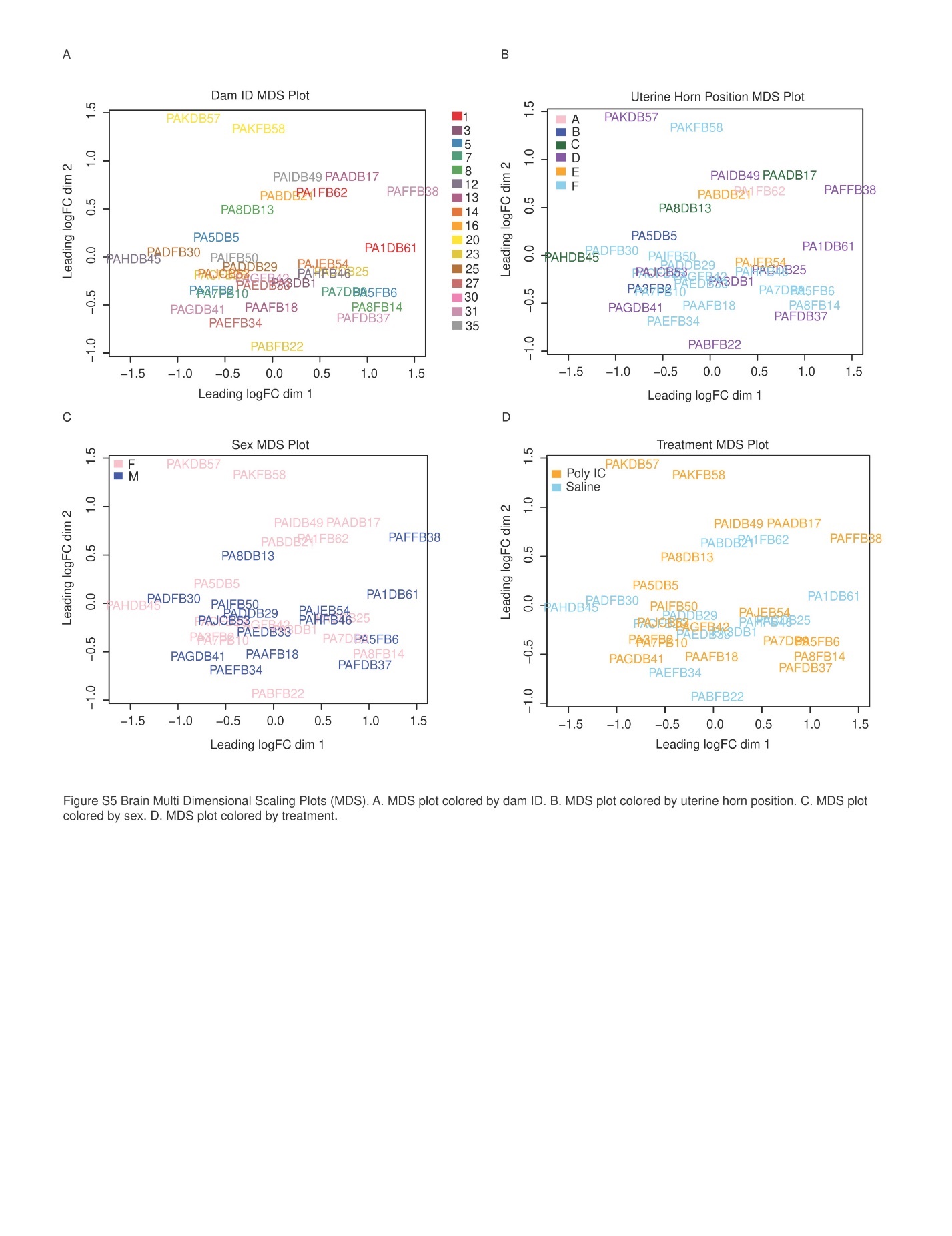

Supplement: Supplementary file 1 — Supplementary Material 1 [file 12974_2024_3106_MOESM1_ESM.docx]
