# Supplementary material for: Impact of maternal immune activation and sex on placental and fetal brain cytokine and gene expression profiles in a preclinical model of neurodevelopmental disorders
Source: J Neuroinflammation. 2024 May 7;21:118. doi: 10.1186/s12974-024-03106-7 (PMC11077729; doi:10.1186/s12974-024-03106-7)
Supplement: Supplementary file 2 — Supplementary Material 2 [file 12974_2024_3106_MOESM2_ESM.docx]

**Supplemental Table Legends**

1. Table S1. All Differentially Expressed Genes (DEG) for All Conditions and differentially expressed genes by sex and tissue.
   1. Sample information: sample ID, treatment, tissue, dam, uterine horn position and sex.
   2. All DEG: Master output file for all genes in the experiment, significance for each comparison, and membership to brain or placenta.
   3. Male placenta DEG.
   4. Male brain DEG.
   5. Female placenta DEG.
   6. Female brain DEG.
2. Table S2: Gene Ontology (GO) and Kyoto Encyclopedia of Genes and Genomes (KEGG) Pathway Analysis for DEG.
   1. GO and KEGG terms for decreased placental DEG.
   2. GO and KEGG terms for increased placental DEG.
   3. GO and KEGG terms for decreased female placental DEG.
   4. GO and KEGG terms for increased female placental DEG.
   5. GO and KEGG terms for decreased male placental DEG.
   6. GO and KEGG terms for increased male placental DEG.
   7. GO and KEGG terms for increased female brain DEG.
